# The Effects of Tobacco

**Published:** 1879-08

**Authors:** 


### THE EFFECTS OF TOBACCO.

My own experience of the evil effects of great tobacco smoking and chewing, is that these are among the most prevalent causes of chronic disease in the male sex. Of course, I do not mean for one moment to compare the dangers caused by the use of tobacco with those we are so familiar with at the bedside, in cases of diseases caused by alcohol. Tobacco does not cause cirrhosis of the liver, nor disease of the lungs and and heart, in the same way, or with the same frequency as chronic tippling does. But there are, nevertheless, several well marked diseases caused by the taking in of the nicotine into the blood, whether through the absorbents of the mouth in smoking, or, more rapidly, in the case of chewing. First of all, the digestive organs are often greatly impaired by the use of *nicotiana tabacum.* The teeth are frequently blackened, and the gums swollen in great smokers and chewers. Caries of the teeth is favored by the various acids produced by the burning of tobacco, and mingled with the saliva. Duskiness of the fauces, and relaxed sore throat, are far too prevalent among smokers, as good observers have long noticed. Dyspepsia, caused by nicotine, is so common as to be hardly worth referring to. Diarrhea, or more frequently constipation, is induced by the use of tobacco, in many instances. And I must not omit, in passing, the remark that the male sex who smoke are alone, with the very rarest exceptions, the subjects of epithelioma of the lip. I once saw such a case in an old Irishwoman, who was a constant pipe-smoker.

With regard to the nervous system, the weakness of vision produced by nicotine is a constant trouble to youthful smokers. Mr. George Critchett has remarked that among wealthy young men, weak sight is very frequently indeed caused by their extravagant addiction to cigars and pipes. Tobacco amaurosis, too, is far from rare. In very young men, the use of nicotine is peculiarly inimical to intellectual improvement. Thus, M. Joly found that in the Polytechnic School at Paris the non-smoking students carried off the very great majority of the prizes for mathematics; and Dr. Kostral, physician to the State factory of tobacco of Austria, shows how nicotine poisoning often kills the young boys of the factory, and causes abortions in the young mothers, and death of infants at the breast, through the nicotine contained in the mother's milk.—*Dr. Drysdale, in Medical Press and Circular.*

